# Review of the State of Impurity Occurrences and Impurity Removal Technology in Phosphogypsum

**DOI:** 10.3390/ma16165630

**Published:** 2023-08-15

**Authors:** Xu Li, Xinfeng Lv, Lan Xiang

**Affiliations:** Department of Chemical Engineering, Tsinghua University, Beijing 100084, China; lixu21@mails.tsinghua.edu.cn (X.L.); lxf19@mails.tsinghua.edu.cn (X.L.)

**Keywords:** phosphate fertilizer, phosphogypsum, impurities, occurrence state, harmless

## Abstract

A variety of co-existing impurities in phosphogypsum limit its large-scale and high-value utilization. This paper summarizes the common contents of major impurity components (silicon and phosphorus) and trace impurity components (fluorine, iron, aluminum, and carbon) in phosphogypsum and discusses the harm of impurity components to the comprehensive utilization of harmless phosphogypsum chemical resources. The occurrence status of impurity components in phosphogypsum and the research progress of various impurity removal technologies are summarized, and the effects of these impurity removal technologies on different contents of impurity components are evaluated. On this basis, the goal of improving the whiteness of phosphogypsum samples and the development of technology for further removal of impurities in phosphogypsum to improve the purity of the main content of calcium sulfate are speculated.

## 1. Introduction

China is the world’s largest phosphate fertilizer producer and has become the world’s largest phosphogypsum resource by-product country, with annual production in phosphogypsum of about 75 million tons, but the comprehensive utilization rate is about 45% only; as the accumulated stockpile has reached 830 million tons, which takes up a lot of land, there is a serious risk of environmental pollution [1,2,3,4] and a waste of precious resources of calcium and sulfur. The main component of phosphogypsum is calcium sulfate dihydrate. Calcium sulfate itself is an excellent cementing material and is rich in calcium and sulfur resources, which can be used as an artificial building material [5,6,7,8,9,10], a subgrade material [11,12,13,14], a backfill material [15], a ceramic material [16], and in the synthesis of new materials (such as nano-hydroxyapatite [17,18,19,20], nano-calcium carbonate [21,22], and calcium sulfate whisker [23,24,25]). It can also be used as a cement retarder [26,27,28], a water purification agent for adsorption of heavy metals [29,30,31,32,33,34], a soil conditioner [35,36,37,38,39,40], a slow-release nitrogen fertilizer [41,42], an agent in the coproduction of sulfuric acid for cement making [43,44], an asphalt improver [45,46,47,48], a plastic filler [49,50,51], an agent in carbon dioxide mineralization sequestration [52,53,54,55], a rare earth raw material [56,57,58], and a material in the preparation of white textured paint [59]. Calcium sulfate dihydrate in phosphogypsum contains a variety of co-existing impurities, such as inorganic and organic silicon, phosphorus, fluorine, iron, aluminum, and carbon, which limits the large-scale and high-value utilization of phosphogypsum [60]. Therefore, reducing the content of impurity elements in phosphogypsum, increasing the main content of calcium sulfate, and improving the harmless comprehensive utilization rate of phosphogypsum resources are of great significance to the sustainable and healthy development of the phosphorus chemical industry. In recent years, research on the occurrence state of various impurity elements in phosphogypsum and impurity removal technologies has become one of the most popular research topics among scientists and technological workers [61,62,63,64,65,66,67,68,69,70,71,72]. According to the contents of various elements in typical phosphogypsum in China, the scope of this review focuses on major impurity components (wt% > 1%, including silicon and phosphorus) and trace impurity components (wt% 0.01–1%, including iron, aluminum, and carbon) in phosphogypsum, excluding other minor trace and elemental impurity components (wt% < 0.01%, such as heavy metals, rare earth elements, and radioactive elements). In this paper, the contents of impurities in typical phosphogypsum are reviewed, and the hazard characteristics of these impurities on the harmless comprehensive utilization of phosphogypsum resources, the phase occurrence state of impurity elements in phosphogypsum, and existing impurity removal technologies are summarized.

## 2. Composition and Occurrence State of Impurities in Phosphogypsum

### 2.1. Elemental Composition of Typical Phosphogypsum

China is rich in phosphate rock resources, ranking second in the world in reserves, which are mainly distributed in Yunnan, Guizhou, Sichuan, and Hubei provinces. The elemental composition of phosphogypsum by-products of typical phosphorus chemical enterprises in these provinces is shown in Table 1.

Divided according to the content of each impurity element, the mass contents of silicon and phosphorus oxides in phosphogypsum are greater than 1%; thus, these two elements have a constant content and are called constant impurity components. The mass contents of fluorine, iron oxide, aluminum oxide, and carbon are between 0.01 and 1%; thus, they have a trace content and are called trace impurity components. Magnesium mostly becomes a phosphoric acid solution during the acid hydrolysis of phosphate rock. The mass content of magnesium oxide in phosphogypsum is usually less than 0.01%, and the contents of other elements, such as heavy metal elements, rare earth elements, and radioactive elements, are also often less than 0.01%; thus, these elements have a trace or an ultra-trace content and are not included in the scope of discussion of this paper on the phase occurrence state and existing impurity removal technologies.

Occurrence state analysis examines the occurrence state of an element in minerals. The occurrence states of elements in minerals mainly include independent minerals, isomorphism, ion adsorption states, and inclusion states. For a certain impurity element in phosphogypsum, multiple mineral occurrence forms can exist in phosphogypsum. The occurrence forms of various impurities in phosphogypsum are an important factor restricting the impurity removal process, which directly affects the purity and application performance of calcium sulfate purification.

### 2.2. Hazard and Occurrence State of Constant Impurity Components

The major impurity components in phosphogypsum include two elemental impurities, silicon and phosphorus. The average content of silicon dioxide in typical phosphogypsum is 7.06%, and the average content of phosphorus pentoxide is 1.17%.

#### 2.2.1. Silicon Impurities

Ma [94] pointed out that the high hardness of quartz sand will cause equipment wear and tear. Although it is not harmful to conventional gypsum products, it affects the purity of gypsum and affects high-end applications, such as high-purity fillers.

Tang et al. [74] pointed out that silicon impurities in phosphogypsum mainly exist in the form of quartz (SiO_2_) and as a small amount of sodium fluorosilicate and potassium fluorosilicate. Among them, quartz impurities exist by themselves, and sodium fluorosilicate and potassium fluorosilicate are distributed between calcium sulfate crystals, which are bonded to each other. Li et al. [86] found that silicon in phosphogypsum mainly exists in the form of quartz using XPS analysis. Huang et al. [95] identified the main form of silicon in phosphogypsum as quartz. Xu et al. [96] found that sodium fluorosilicate exists as insoluble silicon in phosphogypsum.

Zhu et al. [97] revealed that quartz and gypsum mainly exist in the form of monomers, except for a small amount of quartz particles that are wrapped by secondary phosphogypsum, and rarely cemented with each other. Using energy-dispersive spectroscopy (EDS), Pang et al. [92] showed that a small amount of quartz adheres in phosphogypsum crystals in the form of flashpoints. Du et al. [83] reported that quartz in phosphogypsum exists as granular or aphanitic aggregates, with a size of 0.05–0.15 mm and a content of about 6%. Zhang et al. [78] found that the main impurity minerals in their sample of phosphogypsum are quartz and potassium feldspar, accounting for 4.14% and 1.76%, respectively, and their removal is the key to improving the purity of the sample gypsum. Therefore, the main impurity phases of silicon in phosphogypsum include quartz, sodium fluorosilicate, and potassium fluorosilicate. Quartz is the main impurity in phosphogypsum and is an independent mineral. Sodium fluorosilicate and potassium fluorosilicate are insoluble fluorine and exist in a state of inclusion with gypsum. The occurrence state of silicon impurities is shown in Table 2.

#### 2.2.2. Phosphorus Impurity

Li et al. [98] pointed out that insoluble phosphorus does not have a significant impact on the quality of gypsum products. Liu et al. [99] found that soluble phosphorus and eutectic phosphorus make the setting time of phosphogypsum-based products longer, the product structure looser, and the product strength lower. Ma [94] showed that soluble phosphorus causes frost and powdering on the surface of dried gypsum products. Li et al. [100] prepared semi-hydrated gypsum from phosphogypsum at a calcination temperature of 120 °C and found that calcination does not reduce the phosphorus content in phosphogypsum. In the industrial production of semi-hydrated gypsum, pre-treatment is required to reduce the adverse effects of phosphorus impurities. Huang et al. [101] pointed out that when phosphogypsum is used as an additive to calcium sulphoaluminate cement, the existence of phosphate and phosphoric acid impurities in phosphogypsum reduces the compressive strength of cement products.

Zhang et al. [78] reported that phosphorus in phosphogypsum mainly exists as insoluble phosphorus, with a content of 0.55%, while the content of soluble phosphorus is only 0.17%. Due to the immature detection technology of eutectic phosphorus, the content of eutectic phosphorus cannot be detected separately. Tang et al. [74] found that phosphorus impurities in phosphogypsum include aluminum phosphate, fluorophosphoric acid, calcium phosphate, dicalcium phosphate, and monocalcium phosphate via electron backscatter diffraction (EBSD)–X-ray photoelectron spectroscopy (XPS) analysis. Jiang et al. [102] reported that phosphorus in phosphogypsum mainly exists in gypsum in the form of phosphate, with a small amount existing in calcium fluorophosphoric acid, via the use of solid P-31 nuclear magnetic resonance spectrum for the first time. Li et al. [103] found that the infrared characteristic absorption peak of eutectic phosphorus in the sample they measured through infrared absorption spectroscopy is 839 cm^−1^. Ma [94] pointed out that the main forms of eutectic phosphorus in phosphogypsum are CaHPO_4_ · 2H_2_O and NaHPO_4_.

Using a mineral dissociation analysis system (MLA), Zhang et al. [78] found that the monomer dissociation degree of apatite, an insoluble phosphorus element, in phosphogypsum is 33.22%, with about 42.59% being associated with gypsum and about 9.29% being multiphase wrapped with gypsum quartz composite minerals. Therefore, during deep dephosphorization, it is difficult to directly remove apatite using physical methods, and it needs to be removed using methods such as acid washing. Xu et al. [96] pointed out that insoluble phosphorus exists in coarse gypsum particles, mainly consisting of a small amount of unreacted phosphate rock powder. Through their mineral dissociation analysis system (MLA), Zhang et al. [78] found that the dissociation degree of soluble phosphorus monomers in phosphogypsum is 81.38%, and about 6.92% coexists with gypsum quartz composite minerals, while about 4.41% is polyphase wrapped with gypsum. Therefore, during deep dephosphorization, methods such as water washing can be used for removal. Xu et al. [96] reported that soluble phosphorus is generally adsorbed on the surface of gypsum crystals. Using energy spectrum analysis (EDS), Pang et al. [92] showed that there is a small amount of phosphorus in the flash point, like the adhesion of phosphogypsum crystals.

Zhuo [104] pointed out that the content of eutectic phosphorus decreases with an increase in the granularity of phosphogypsum. Therefore, the main impurity phases of phosphorus elements in phosphogypsum include insoluble phosphorus, soluble phosphorus, and eutectic phosphorus. Insoluble phosphorus includes calcium fluorophosphoric acid, calcium phosphate, iron (III) phosphate, aluminum phosphate dihydrate, and basic calcium phosphate dihydrate. The inclusion state is dominant, followed by independent minerals. Soluble phosphorus consists of soluble salts such as phosphoric acid, dihydrogen phosphate, hydrogen phosphate, and phosphate, which are independent minerals or exist in ion adsorption states. Eutectic phosphorus includes dicalcium phosphate dihydrate, sodium hydrogen phosphate and fluorophosphoric acid dihydrate. A solid solution is formed by replacing SO_4_^2−^ with HPO_4_^2−^ and FPO_3_^2−^ in the calcium sulfate lattice, and it is mainly isomorphic. The occurrence status of phosphorus impurities is detailed in Table 3.

### 2.3. Harm and Occurrence State of Trace Impurity Components

The trace impurities in phosphogypsum include fluorine, iron, aluminum, and carbon. The average content of fluorine in typical phosphogypsum is 0.48%, the average content of ferric oxide is 0.43%, the average content of aluminum oxide is 0.68%, and the average content of carbon is 0.12%. 

#### 2.3.1. Fluorine Impurities

Ding et al. [105] pointed out that when the fluorine content in phosphogypsum is greater than 1.0%, crystal development is affected, the setting time is prolonged, crystal particle size is reduced, and the strength of semi-hydrated gypsum products is significantly reduced. Soluble fluorine in phosphogypsum enters water bodies, pollutes soil, and enters the human body via the ecological food chain, leading to dental fluorosis or skeletal fluorosis, such as tooth softening and osteoporosis. Jia et al. [106] found that in the preparation of phosphogypsum β because sodium fluoride generates calcium fluoride precipitation and soluble sodium sulfate, the 2 h dry strength of hemihydrate gypsum is reduced. Li et al. [15] reported that when phosphogypsum is used as a backfill material, calcium fluoride precipitation is hydrated to release fluoride, and there is a risk that the fluoride content will exceed the Chinese Standard (GB8978-1996) (10 mg/L). Fluorine needs to be removed before use or converted into insoluble fluoride with a lower solubility than calcium fluoride. 

Tang et al. [74] analyzed the impurity phase in phosphogypsum using electron backscatter diffraction (EBSD) and the phase composition using energy-dispersive X-ray spectroscopy (EDS). The fluorine impurity in phosphogypsum exists in two forms: calcium fluoride and fluorophosphoric acid. The X-ray photoelectron spectroscopy (XPS) analysis shows that fluorine impurities in phosphogypsum include sodium fluorosilicate, potassium fluorosilicate, fluorophosphoric acid, calcium fluoride, magnesium fluoride, and aluminum fluoride. Using a combination of X-ray photoelectron spectroscopy (XPS) and electron microprobe (EMPA), Zhao et al. [90] identified that the trace fluoride phase and relative content in phosphogypsum are, respectively, 41.52% of sodium fluorosilicate and potassium fluorosilicate, 30.61% of AlF_2.3_(OH)_0.7_ · H_2_O, 12.21% of sodium hexafluoroaluminate and potassium aluminate, 8.42% of Ca_5_(PO_4_)_3_F and Ca_10_(PO_4_)_6_F_2_, 3.62% of calcium fluoride, 2.41% of AlF_3_ · 3H_2_O, and 1.21% of sodium fluoride and potassium fluoride.

Li et al. [107] pointed out that the content of soluble fluorine in phosphogypsum increases with an increase in gypsum particle size. Peng et al. [108] found that soluble fluorine in phosphogypsum is mainly distributed on the surface of gypsum crystals using SEM/EDXA analysis.

Therefore, the main impurity phases of fluorine elements in phosphogypsum include insoluble fluorine and soluble fluorine, but mainly insoluble fluorine. Insoluble fluorine includes calcium fluoride, sodium fluorosilicate, potassium fluorosilicate, sodium hexafluoroaluminate, potassium aluminate, magnesium fluoride, aluminum fluoride, fluorophosphoric acid calcium, and other fluorophosphates; it mainly exists in the state of inclusion, which is supplemented by the state of isomorphism. Soluble fluorine includes fluoride ions, sodium fluoride, potassium fluoride, hydrofluoric acid, and hexafluorosilicic acid, and the ion adsorption state is dominant. The occurrence status of fluorine impurities is detailed in Table 4.

#### 2.3.2. Iron Impurity

Li et al. [109] pointed out that iron is the highest dyeing metal element in phosphogypsum, which has the greatest impact on the whiteness of phosphogypsum and makes phosphogypsum gray and yellow.

Tang et al. [74] analyzed the impurity phase using electron backscatter diffraction (EBSD) and the phase composition using energy-dispersive X-ray spectroscopy (EDS). The iron impurity in phosphogypsum exists in the form of pyrite (FeS_2_). Pyrite from phosphate rock does not react during acidolysis and is deposited in phosphogypsum. Du et al. [83] pointed out that pyrite (FeS_2_) in phosphogypsum is distributed as fine particles or fine particle aggregates, with a particle size of 0.01–0.10 mm and content of about 1%. Li et al. [81] reported that the main iron in phosphogypsum is amorphous iron, including hematite (Fe_2_O_3_), limonite (Fe_2_O_3_ · H_2_O), goethite [FeO(OH)], and siderite (FeCO_3_). Zhu et al. [97] pointed out that there are trace goethite FeO(OH) and other minerals in phosphogypsum. Iron existing in the mineral lattice in the form of isomorphism is called eutectic iron, and iron existing in iron-containing impurities that do not enter the lattice is called amorphous iron. Li et al. [109] showed that iron impurities in phosphogypsum are mainly distributed as particles with a particle size greater than 0.8 mm.

Therefore, the main impurity phases of iron element in phosphogypsum include amorphous iron and eutectic iron, with amorphous iron being the main impurity phase. Amorphous iron includes ferric disulfide, basic iron (II) oxide, ferric oxide, ferric oxide trihydrate, and ferric carbonate, and the inclusion state is dominant. Eutectic iron includes iron (II) phosphate dihydrate, iron (III) sulfate, iron (II) sulfate, and iron (III) sulfate heptahydrate, which are mainly isomorphic. The occurrence status of iron impurities is detailed in Table 5.

#### 2.3.3. Aluminum Impurities

There is currently no evidence of obvious harm from aluminum impurities, but there is an effect on the purity of the main content of calcium sulfate in products.

By analyzing the impurity phase using electron backscatter diffraction (EBSD) and the phase composition using energy-dispersive X-ray spectroscopy (EDS), Tang et al. [74] found that aluminum impurity in phosphogypsum exists as an Al_2_O_3_ phase. The X-ray photoelectron spectroscopy (XPS) analysis shows that aluminum impurities in phosphogypsum include aluminum oxide, aluminum fluoride, aluminum sulfate, and aluminum phosphate.

Zhao et al. [90] found that the micro-aluminous phase in phosphogypsum includes AlF_2.3_(OH)_0.7_ · H_2_O, sodium hexafluoroaluminate, potassium aluminate, and AlF_3_ · 3H_2_O using a combination of X-ray photoelectron spectroscopy (XPS) with an electron microprobe (EMPA).

Using a combination of electron backscatter diffraction (EBSD), X-ray energy spectrum (EDS), and X-ray photoelectron spectroscopy (XPS), Tang et al. [74] found that the occurrence and relationship of the impurity phase in phosphogypsum are as follows: aluminum oxide is in an aggregated phase in phosphogypsum, while aluminum and other elements form double salt binding distribution, such as aluminum sulfate, aluminum phosphate, and aluminum fluoride.

Therefore, the main impurity phases of aluminum elements in phosphogypsum include oxides and double salts, but mainly oxides. Oxide exists as aluminum oxide, which is an independent mineral. Double salts include aluminum sulfate, aluminum phosphate, hydrated basic aluminum fluoride, trihydrate aluminum fluoride, sodium hexafluoroaluminate, and potassium aluminate, and the inclusion state is dominant. The occurrence status of aluminum impurities is detailed in Table 6.

#### 2.3.4. Carbon Impurity

Laing et al. [76] pointed out that phosphogypsum has a low organic content, a light weight, easy dispersion, and easy adsorption on the surface of phosphogypsum particles, and it can reduce the whiteness of phosphogypsum. Ma et al. [94] showed that organic matter can delay the setting time of building gypsum and reduce the strength of cementitious materials.

Wang et al. [110] pointed out that phosphogypsum contains a certain amount of black particles. Using energy spectrum analysis, they found that the characteristic peaks of carbon are 1360 cm^−1^ and 1580 cm^−1^, indicating that these black particles are mainly carbonaceous. Li et al. [81] reported that carbon in phosphogypsum can be divided into inorganic carbon and organic carbon, and graphite carbon is the main inorganic carbon in phosphogypsum, which has a great impact on whiteness; this is accompanied by a small amount of carbonate inorganic carbon, which has little impact on whiteness. Wang [111] pointed out that macromolecular organic matter in phosphogypsum is closely combined with minerals and various insoluble acids and alkalis. It belongs to humin in soil humic acid. Peng et al. [112] reported that the medium and small molecular organics of phosphogypsum are ethylene glycol methyl ether acetate, isothiomethane, 3-nenenebb methoxy pentane, 2-ethyl-1,3-dioxolane, etc. Organic matter shows flocculent distribution on the surface of gypsum crystals, and its content increases with an increase in gypsum particles.

Wang [111] pointed out that organic matter groups such as humin in phosphogypsum react chemically with unsaturated S in pyrite to form incomplete laminated wrapping between the organic matter and pyrite. Even if excessive hydrochloric acid is used to decompose pyrite in phosphogypsum twice, a certain amount of pyrite can be detected during XRD characterization, indicating that some pyrite is completely wrapped by inert organic matter, such as humin in the outer layer.

Therefore, the main impurity phases of carbon elements in phosphogypsum include inorganic mixed crystals, inorganic single crystals, organic macromolecules, and organic small molecules, with inorganic mixed crystals being the main ones. Inorganic mixed crystals are made of graphite carbon, while inorganic single crystals consist of a small amount of carbonate. Organic macromolecules include humin, humic acid, and fulvic acid, with the inclusion state being the main state. Organic small molecules include ethylene glycol methyl ether acetate, isothiomethane, 3-neneneba methoxy pentane, 2-ethyl-1,3-dioxolane, mineral processing agent, and organic flocculant, with the ion adsorption state being dominant. The occurrence status of carbon impurities is detailed in Table 7.

## 3. Phosphogypsum Removal Impurity Technologies

In view of different elemental impurities in phosphogypsum, and according to the number of elemental content and the characteristics of the occurrence state of impurities, a variety of impurity removal technologies have been developed, which are mainly divided into major component impurity removal technologies and trace component impurity removal technologies according to the amount of each impurity element content.

### 3.1. Major Component Impurity Removal Technology

Silicon and phosphorus are the top two major impurity elements in phosphogypsum, and their impurity removal techniques are either single or combined. Single decontamination techniques include water washing, swirl, flotation, screening, and acid leaching. Combined decontamination techniques include sieve-flotation, acid leaching–oxidation and acid leaching–extraction methods.

#### 3.1.1. Silicon Impurity Removal

Zhu et al. [97] and Tan et al. [113] treated phosphogypsum with a single impurity removal technology (swirl and flotation, respectively) and investigated the removal of silicon impurities. Tan et al. [113] indicated that under the condition of 20% slurry solid content, the cyclone used can realize the grading of phosphogypsum particle size, and the silica content is reduced from 4.26% to 2.79% (the average particle size of the settling mouth is 51.7 µm). Zhu et al. [97] indicated that when phosphogypsum is treated with the “one crude and two fine” method at a pH of 2.0, the silica content in the raw material is reduced from 12.25% to 2.69%. Zhang et al. [80] showed that silicon dioxide in phosphogypsum slurry with a 30% concentration can be reduced from 12.03% to 1.17% by using dodecylamine flotation. Zhang et al. [114] indicated that the flotation of silica in gypsum can be achieved using the surfactant tetracyl-dimethylbenzyl ammonium chloride (TDBAC) in the pH range of 2.5–9.5, and the best concentration of collector is 10^−4^ mol/L.

Zou et al. [73] and Li et al. [115] used combined removal technologies (acid leaching–extraction, acid leaching–oxidation, sieve-flotation, and reverse flotation–positive flotation methods) to treat phosphogypsum and investigated the removal of silicon impurities. Li et al. [115] indicated that the content of silica in phosphogypsum can be reduced to 0.12% by using 30% sulfuric acid hydrolysis coupled with tributyl phosphate as an organic solvent. Zou et al. [73] indicated that silicon dioxide in phosphogypsum can be reduced from 5.99% to 3.54% by using a treatment combining 20% sulfuric acid and 20% hydrogen peroxide. Zou et al. [116] indicated that the silica content in the raw material can be reduced from 5.59% to 1.55% via flotation using dodecylamine at a pH of 3.0. Guo et al. [117] indicated that after the removal of −0.0308 mm fine-size gypsum through pre-screening and grading at a pH of 7.0, the silicon dioxide content in the raw material can be reduced from 11.74% to 0.18% using a self-made flotation collector with dodecamine as the main component. Wei et al. [75] reported that the silica content in phosphogypsum can be reduced from 7.84% to 1.56% by using emulsifier reverse flotation and a self-made collector at a pH of 1.5.

Zhao et al. [88] compared the removal of silicon impurities via the use of single and combined impurity removal techniques. They indicated that silicon dioxide in raw materials can be reduced from 5.82% to 5.21%, 3.15%, and 0.12%, respectively, using water washing, pickling, and pickling coupled with solvent extraction.

It can be seen that single impurity removal technologies can remove silicon impurities close to 2.05% of the content, and most of the combined impurity removal technologies can remove silicon impurities in the trace content range at about 0.15%.

#### 3.1.2. Phosphorus Impurity Removal

Pang et al. [92] and Tan et al. [113] treated phosphogypsum with single impurity removal technologies (screening, swirling, and acid leaching) and investigated the removal of phosphorus impurities. Pang et al. [92] indicated that after screening, the content of total phosphorus impurities in phosphogypsum decreases with a decrease in particle size of phosphogypsum. The phosphorus dioxide content of phosphogypsum is 1.58%, which is less than 46, and the total phosphorus content of phosphogypsum is less than 0.68%. Peng et al. [112] found that the total phosphorus content of phosphogypsum can be reduced from 1.75% to 1.06% by removing coarse particles larger than 200 µm through screening. Tan et al. [113] indicated that under the condition of 20% solid content of slurry, the particle size of phosphogypsum can be graded by the cyclone, and the content of phosphorus pentoxide can be reduced from 1.03% of the raw material to 0.79% (the average particle size of the settling mouth is 59.4 µm). Li et al. [84] reported that after leaching phosphogypsum with 30% sulfuric acid at 55 °C for 120 min, the characteristic peak of eutectic phosphorus at 839 cm^−1^ disappears, and P_2_O_5_ in the raw material decreases from 0.82% to 0.008%.

Wang et al. [85] and Guo et al. [117] treated phosphogypsum using combined impurity removal technologies (sieving and flotation, reverse flotation, and forward flotation) and investigated the removal of phosphorus impurities. Guo et al. [117] indicated that after the removal of –0.0308 mm fine-grade gypsum through pre-screening and grading at a pH of 7.0 and with dodecamine being used as the main component in the self-made flotation collector, the phosphorus pentoxide content in the raw material changes from 1.10% to 1.20%, indicating that flotation has no obvious effect on the removal of total phosphorus. Wang et al. [85] indicated that phosphogypsum is deslimed by methyl isobutyl methanol reverse flotation, followed by dodecylamine “one coarse, one clean and two fine” closed-circuit flotation and the phosphorus pentoxide content in gypsum decreases from 1.78% to 0.92%. Wei et al. [75] indicated that phosphorous pentoxide in raw materials can be reduced from 0.968% to 0.803% by using emulsifier reverse flotation and a self-made collector at a pH of 1.5. Qi et al. [118] indicated that phosphorus pentoxide in phosphogypsum is reduced from 1.44% to 0.046% after using reverse flotation of silanol and positive flotation of dodecylamine hydrochloride.

Zhao et al. [88] compared the removal of phosphorus impurities using single and combined impurity removal techniques. They found that P_2_O_5_ in raw materials can be reduced from 0.79% to 0.46%, 0.02%, and 0.01%, respectively, using water washing, pickling, and pickling coupled solvent extraction.

It can be seen that both single and combined impurity removal techniques can basically remove phosphorus impurities in phosphogypsum to a trace range (<1%).

### 3.2. Microcomponent Impurity Removal Technology

The contents of fluorine, iron, aluminum, carbon, and other elements in phosphogypsum are in the trace range (between 0.01 and 1%), and the impurity removal methods of these elements have gradually changed from using a single impurity removal technology to using a combined impurity removal technology.

#### 3.2.1. Fluoride Impurity Removal

Li et al. [84] and Peng et al. [93] treated phosphogypsum using single impurity removal technologies (screening, washing, lime neutralization, and acid leaching) and investigated the removal of fluorine impurities. Peng et al. [112] indicated that the content of soluble fluorine in phosphogypsum can be reduced from 0.50% to 0.37% by screening and removing coarse particles with a size > 200 μm. Peng et al. [93] showed that the soluble fluorine content in phosphogypsum can be reduced from 0.50% to 0% via washing and lime treatment. Among these techniques, the washing condition involves washing phosphogypsum with three times the amount of tap water 3–4 times until the washing liquid is neutral; the condition of lime neutralization involves adding lime to phosphogypsum with a water content of about 15%, mixing well, and then aging for 24 h. Li et al. [84] indicated that the content of soluble fluorine in phosphogypsum is reduced from 0.02% to 0.014% after aging with 0.4% lime for 12 h. They showed that through the use of 30% sulfuric acid, with a liquid/solid ratio of 3 mL/g, a temperature of 55 °C, and acid leaching for 120 min, the total fluorine content in phosphogypsum can be reduced from 0.18% to 0.016%. Yang [77] indicated that with 30–35% sulfuric acid leaching at 90 °C, the total fluorine content in phosphogypsum can be reduced from 0.23% to 0.06%.

Peng et al. [93] and Li et al. [115] investigated the removal of fluorine impurities in phosphogypsum by using combined removal technologies (neutralization–calcination, acid leaching–extraction, acid leaching–oxidation, and screening–flotation). Jiahui Peng et al. [93] reported that the content of soluble fluorine in phosphogypsum can be reduced from 0.50% to 0% via neutralizing calcination. The condition of neutralizing calcination involves phosphogypsum that has been neutralized by lime being calcined at 800 °C for 2 h. Li et al. [115] reported the extraction of phosphogypsum using 30% sulfuric acid hydrolysis coupled with tributyl phosphate, and the fluorine content in gypsum can be reduced to be lower than the detection limit. Zou et al. [73] showed that the total fluorine content in phosphogypsum can be reduced from 0.86% to 0.10% by using a treatment combining 20% sulfuric acid and 20% hydrogen peroxide. Kai Zou [116] indicated that the fluorine content in raw materials can be reduced from 0.1% to 0.06% through the flotation of dodecylamine at a pH of 3.0.

Zhang et al. [80] and Zeng et al. [119] compared the removal of fluorine impurities using single and combined impurity removal techniques. Zeng et al. [119] indicated that the water-soluble fluorine content in phosphogypsum can be reduced from 0.34% to 0.11%, 0.10%, and 0.077%, respectively, via washing, neutralization, or calcination. Zhang et al. [80] showed that the soluble fluorine content in phosphogypsum can be reduced from 0.250% to 0.023%, 0.02%, and 0.018%, respectively, when treated with lime neutralization, positive flotation, and water washing. Zhao et al. [88] indicated that after water washing, pickling, and pickling coupled with solvent extraction, the fluorine content of raw materials can be reduced from 0.87% to 0.61%, 0.27%, and no trace amounts, respectively.

In conclusion, aside from the poor fluorine removal effect of screening and washing, other single and combined impurity removal technologies have better removal effects on fluorine impurities in phosphogypsum (fluorine content after impurity removal ranges from 0 to 0.3%).

#### 3.2.2. Iron Impurity Removal

Yang [77] treated phosphogypsum using a single impurity removal technique (acid leaching) and investigated the removal of iron impurities. He found that the ferric oxide content in phosphogypsum can be reduced from 0.28% to 0.21% by using 30–35% sulfuric acid and acid leaching at 90 °C.

Li et al. [115] and Zou et al. [116] investigated the removal of iron impurities by treating phosphogypsum with combined techniques (sifting–flotation, acid leaching–extraction, acid leaching–oxidation, and reaction–calcination). Zou et al. [116] found that the ferric oxide content in the raw material can be reduced from 0.32% to 0.13% using the flotation of dodecylamine at a pH of 3.0. Li et al. [115] indicated that the content of ferric oxide in phosphogypsum can be reduced to 0.02% by using 30% sulfuric acid hydrolysis coupled with tributyl phosphate as an organic solvent. Zou et al. [73] indicated that the ferric oxide content in phosphogypsum can be reduced from 0.13% to 0.02% by combining 20% sulfuric acid and 20% hydrogen peroxide. Fang et al. [120] reported that by adding a reaction with 2% ammonium chloride, at a calcination temperature of 475 °C and a calcination time of 2 h, in the treatment of phosphogypsum, the ferric oxide content can be reduced from 0.38% to 0.012%.

Zhao et al. [88] compared the removal of iron impurities using single and combined impurity removal techniques. They indicated that the content of ferric oxide in raw materials can be reduced from 0.51% to 0.46%, 0.29%, and 0.02%, respectively, using water washing, pickling, and pickling coupled solvent extraction.

It can be seen that, at present, the best removal effect of single or combined impurity removal technologies can only achieve a reduction of iron trioxide content to 0.02% (about 200 ppm) in phosphogypsum.

#### 3.2.3. Aluminum Impurity Removal

Yang [77] and Tan et al. [113] treated phosphogypsum using single impurity removal technologies (screening and acid leaching, respectively) and investigated the removal of aluminum impurities. Tan et al. [113] indicated that under the condition of 20% slurry solid content, the cyclone can realize the grading of phosphogypsum particle size, and the alumina content is reduced from 0.25% to 0.17% (the average particle size of the settling mouth is 59.4 µm). Yang [77] found that by using 30–35% sulfuric acid and acid leaching at 90 °C, the alumina content in phosphogypsum can be reduced from 0.20% to 0.056%.

Li et al. [115] studied the removal of aluminum impurities by treating phosphogypsum with a combined technology (acid leaching and extraction). They showed that the content of alumina in phosphogypsum can be reduced to 0.02% by using 30% sulfuric acid hydrolysis coupled with tributyl phosphate as an organic solvent.

It can be seen that because aluminum impurities in phosphogypsum present no obvious harm to the comprehensive utilization of the harmless chemical resources of phosphogypsum, the removal of aluminum impurities is of less concern, and the content of aluminum impurities can generally be reduced to less than 0.08%.

#### 3.2.4. Carbon Impurity Removal

Liang et al. [76] and Tan et al. [113] studied the removal of carbon impurities in phosphogypsum using a single technology (swirl and water washing, respectively). Tan et al. [113] indicated that under the condition of 20% solid content of slurry, the cyclone can realize the grading of phosphogypsum particle size, and the organic content is reduced from 0.99% to 0.30% (the average particle size of the settling mouth is 51.7 µm). Liang et al. [76] found that the organic matter content in phosphogypsum can be reduced from 0.24% to 0.13% after washing three times.

Fang et al. [120] studied the removal of carbon impurities in the treatment of phosphogypsum using a combined technology (reaction–calcination). They reported that by adding a reaction with 2% ammonium chloride, at a calcination temperature of 475 °C and a calcination time of 2 h, in the treatment of phosphogypsum, the organic matter content can be reduced from 0.67% to 0.37%.

Peng et al. [93] compared the removal of carbon impurities using single and combined impurity removal techniques. They found that the content of organic matter in phosphogypsum can be reduced from 0.12% to 0.08% and 0% using sieving and calcination, respectively. The screening condition involves removing coarse particles of phosphogypsum > 200 μm. The condition of neutralization calcination involves phosphogypsum, after neutralization with lime, being calcined at 800 °C for 2 h.

It can be seen that at present, the removal effect of carbon impurities, an important factor affecting the whiteness of phosphogypsum, can only be reduced to 0.08% (about 800 ppm) using a single or combined removal technology, and it needs to be calcined at 800 °C for a long time with high energy consumption to be completely removed.

## 4. Conclusions

The studies were conducted by other authors because the article is a review paper. Many studies have been conducted on the impurity removal methods of major impurity components (silicon and phosphorus) and trace impurity components (fluorine, iron, aluminum, and carbon) in phosphogypsum, and a variety of single or combined impurity removal methods have been developed. Many single impurity removal methods have been widely used in industry, such as screening, swirling, washing, lime neutralization, acid leaching, and calcination, and their impurity removal effect is general. There are also some single impurity removal methods that have been studied in depth, such as flotation and oxidation, and their impurity removal effect has been improved to a certain extent. Many new combined impurity removal methods are being researched in the laboratory at a developmental stage, and there is enthusiasm about their industrial application. Currently, the combined impurity removal methods under development mainly include acid leaching–extraction, acid leaching–oxidation, acid leaching–flotation, screening–flotation, neutralization–calcination, and reaction–calcination methods, and their impurity removal effect has been further improved. The combined removal methods at the laboratory stage can all reduce the impurity content to the trace range (<0.7%).

The existing single or combined impurity removal methods have been used to remove the main impurity components (phosphorus, silicon, fluorine, iron, aluminum, and carbon) in phosphogypsum; although the impurity content is reduced to the trace range (between 0.01 and 1%), the total amount of various impurity components is still large and, thus, still restricts the main content of calcium sulfate during purity improvement. It is one of the future development directions to develop a combined impurity removal technology with better selectivity and stronger impurity removal ability. In previous research work, the iron impurity content in phosphogypsum could be removed to about 200 ppm, and the carbon impurity content could be removed to about 800 ppm; according to the paint and food industries’ color control standards and practices, the content of iron impurity in a sample should be ≤30 ppm and the carbon impurity content should be ≤80 ppm. These are two elemental impurities that do not have a significant impact on the whiteness of purified gypsum samples. The deep removal of iron and organic matter is the key bottleneck for improving the added value of phosphogypsum and the focus of future new technology development.

## Figures and Tables

**Table 1:** Elemental composition of typical phosphogypsum in China.

No.	Main Content/%		Constant Impurity Components/%		Trace Impurity Components/%				Ref.
	CaO	SO_3_	SiO_2_	P_2_O_5_	F	Fe_2_O_3_	Al_2_O_3_	C	
1	41.29	49.77	5.99	0.94	0.86	0.13	0.67	0.04	Zou et al. [73]
2	40.73	52.2	2.54	2.39	0.93	0.21	0.5		Tang et al. [74]
3	34.8	53.79	7.84	0.97		0.68	0.9		Wei et al. [75]
4	32.93	42.4	4.26	1.03	0.5	0.47	0.25	0.24	Liang et al. [76]
5	31.32	44.2	2.39	1.36	0.23	0.28	0.2		Yang et al. [77]
6	30.99	45.06	5.43	0.66		0.25	0.56		Zhang et al. [78]
7	28.91	39.7	7.41	1.25	0.29	0.41	0.94		Gu et al. [79]
8	28.19	36.42	12.03	0.92	0.32	0.14	0.74		Zhang et al. [80]
9	25.8	35.17	12.92	0.95		0.15	0.08	0.03	Li et al. [81]
10			9.84	1.19	0.54	0.13	0.28		Zhou [82]
11	41.31	49.33	5.03	1.49		0.58	1.42		Du et al. [83]
12	34.07	40.09	5.29	0.82	0.18	0.2	0.13	0.02	Li et al. [84]
13	39.47	49.03	3.68	1.78	0.06	1.95	2.59		Wang et al. [85]
14	35.93	51.95	8.42	1.51	0.42	0.54	0.52		Li et al. [86]
15	28.63	32.2	17.06	0.7	0.52	0.21	0.24		Li et al. [87]
16	34.46	55.95	5.82	0.79	0.87	0.51	0.66		Zhao et al. [88]
17	31.98	45.42	14.61	0.96		0.15	1.68	0.25	Wu et al. [89]
18	33.64	56.92	6.3	0.7	0.91	0.33	0.64		Zhao et al. [90]
19	34.03	41.7	7	0.18		0.39			Shi et al. [91]
20	32.05	44.32	5.49	1.75	0.38	0.33	0.4	0.12	Pang et al. [92]
21	30.34	43.9	2.11	1.58	0.24	0.24	0.31	0.08	Pang et al. [92]
22	31.6	45.9	3.81	1.75	0.5	1.22	0.62	0.12	Peng et al. [93]
Avr	**33.45**	**45.5**	**7.06**	**1.17**	**0.48**	**0.43**	**0.68**	**0.12**	

**Table 2:** Occurrence status of silicon element impurities.

Impurity Phase	Occurrence Status	Remarks
SiO_2_	Independent mineral	Main impurities
Na_2_SiF_6_/K_2_SiF_6_	Independent mineral	Slightly soluble

**Table 3:** Occurrence status of phosphorus impurities.

Impurity Phase	Occurrence State	Remarks
Ca_5_(PO_4_)_3_F, Ca_3_(PO_4_)_2_, FePO_4_, AlPO_4_·2H_2_O, and Ca_3_(PO_4_)_2_(OH)·2H_2_O	Inclusion state is dominant Independent minerals take second place	Slightly soluble phosphorus
H_3_PO_4_, H_2_PO_4_^−^, HPO_4_^2−^, PO_4_^3−^	Independent mineral or ion adsorption state	Soluble phosphorus
CaHPO_4_·2H_2_O, NaHPO_4_, and CaFPO_3_·2H_2_O	Primarily isomorphic	Eutectic phosphorus

**Table 4:** Occurrence status of fluorine impurities.

Impurity Phase	Occurrence State	Remarks
CaF_2_, Na_2_SiF_6_, K_2_SiF_6_, Na_3_AlF_6_, K_3_AlF_6_, MgF_2_, AlF_3_, Ca_5_(PO_4_)_3_F, CaFPO_3_·2H_2_O, and other fluorophosphates	Inclusion state is dominant Isomorphism as auxiliary	Slightly soluble fluorine
F^−^, NaF, KF, HF, and H_2_SiF_6_	Mainly in ion adsorption state	Soluble fluorine

**Table 5:** Occurrence states of iron impurities.

Impurity Phase	Occurrence State	Remarks
FeS_2_ (yellow), FeO(OH) (yellow), Fe_2_O_3_ (reddish brown), Fe_2_O_3_·3H_2_O (ocher), and FeCO_3_ (grey white)	Inclusion state is dominant	Amorphous intergranular iron
FePO_4_·2H_2_O (white), Fe_2_(SO_4_)_3_ (light yellow), FeSO_4_ (light green), and Fe_2_(SO_4_)_3_·7H_2_O (light yellow)	Primarily isomorphic	Eutectic iron

**Table 6:** Occurrence status of aluminum impurities.

Impurity Phase	Occurrence State	Remarks
Al_2_O_3_	Independent mineral	Oxide
Al_2_(SO_4_)_3_, AlPO_4_, AlF_2.3_(OH)_0.7_·H_2_O, AlF_3_·3H_2_O, Na_3_AlF_6_, and K_3_AlF_6_	Inclusion state is dominant	Double salt

**Table 7:** Occurrence status of carbon impurities.

Impurity Phase	Occurrence State	Remarks
Graphite carbon	\	Inorganic mixed crystals
Carbonate carbonaceous	\	Inorganic single crystals
Humin, humic acid, and fulvic acid	Inclusion state is dominant	Organic macromolecules
Ethylene glycol methyl ether acetate, isothiomethane, 3-neneneba methoxy Pentane, 2-ethyl-1, 3-dioxolane, mineral processing agent, and organic flocculant	Mainly in ion adsorption state	Organic small molecules

## Data Availability

Not applicable.
